# Analysis of repetitive element expression in the blood and skin of patients with Parkinson’s disease identifies differential expression of satellite elements

**DOI:** 10.1038/s41598-019-40869-z

**Published:** 2019-03-13

**Authors:** Kimberley J. Billingsley, Freddy Lättekivi, Anu Planken, Ene Reimann, Lille Kurvits, Liis Kadastik-Eerme, Kristjan M. Kasterpalu, Vivien J. Bubb, John P. Quinn, Sulev Kõks, Pille Taba

**Affiliations:** 1 Department of Molecular and Clinical Pharmacology, Institute of Translational Medicine, University of Liverpool, Liverpool, UK; 2 Department of Pathophysiology, University of Tartu, Tartu, Estonia; 3 Department of Neurology, University of Tartu, Tartu, Estonia; 4 Faculty of Medicine, University of Tartu, Tartu, Estonia; 5 Estonian Defense forces, Tartu, Estonia; 6 Perron Institute for Neurological and Translational Science, Sarich Neuroscience Research Institute, 8 Verdun St, Nedlands, 6009 Western Australia Australia; 7 Centre for Comparative Genomics, Murdoch University, Murdoch, 6150 Western Australia Australia

## Abstract

Repetitive elements (RE) constitute the majority of the human genome and have a range of functions both structural and regulatory on genomic function and gene expression. RE overexpression has been observed in several neurodegenerative diseases, consistent with the observation of aberrant expression of RE posing a mutagenic threat. Despite reports that associate RE expression with PD no study has comprehensively analysed the role of these elements in the disease. This study presents the first genome-wide analysis of RE expression in PD to date. Analysis of RNA-sequencing data of 12 PD patients and 12 healthy controls identified tissue-specific expression differences and more significantly, differential expression of four satellite elements; two simple satellite III (repName = CATTC_n and _GAATG_n) a high-copy satellite II (HSATII) and a centromeric satellite (ALR_Alpha) in the blood of PD patients. In support of the growing body of recent evidence associating REs with neurodegenerative disease, this study highlights the potential importance of characterization of RE expression in such diseases.

## Introduction

Parkinson’s disease (PD) is the second most common progressive neurodegenerative condition^1^, with late-onset forms of PD affecting around 3–4% of individuals over the age of 80^2,3^. It is characterized pathologically by the degeneration and loss of the dopaminergic neurons in the substantia nigra and the presence of a-synuclein aggregates called Lewy bodies in the central nervous system (CNS)^3,4^. However recent studies have associated more widespread involvement of other CNS structures and peripheral tissue with the disease etiology^5–9^.

Despite huge successes in identifying genetic mutations and risk factors associated with PD, to date there has been little success in developing definitive diagnostic and prognostic biomarkers for the disease. The only definitive diagnosis for PD is performed post-mortem. As onset of the molecular and cellular neuropathology seen in PD likely initiates decades before the manifestation of the motor symptoms, the need for developing a diagnostic marker in readily available tissue is urgent, not only for early intervention but also to monitor progression of therapeutic treatments^10^. Recent efforts have focused on identifying biomarkers of PD in peripheral tissues, with studies identifying molecular alterations in the blood and skin of PD patients. Notably the transcriptional profile from the blood and skin of PD patients demonstrated dysregulation of genes known to be associated with PD^11–13^.

Repetitive element (RE) sequence constitutes the majority of the human genome. Recently the field have seen the development of more sophisticated bioinformatic methods, that can now accurately analyze RE expression ans this has led to the role of RE in disease etiology becoming increasingly apparent. RE can be broadly split into five categories each of which, have very distinct functions. The first four minor categories account for ~10% of the genome, and include; simple sequence repeats, segmental duplications, tandem repeats and satellite DNA sequences, and processed pseudogenes. The fifth and most major and abundant group of RE are transposable elements (TE)s^14^.

**Figure 1:**
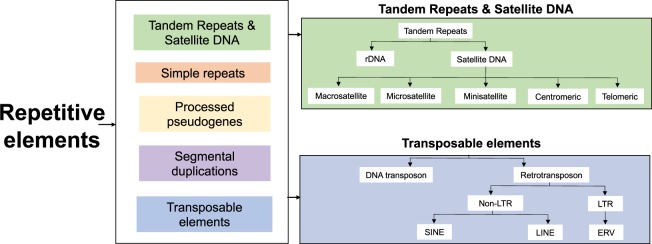
Repetitive DNA classes in the human genome. The major class of RE are Transposable elements, which can be further divided into DNA transposons or Retrotransposons according to there mechanism of transposition, i.e through a RNA or DNA intermediate. Retrotransposons are the most abundant class in the human genome and can be further divided into long terminal repeats (LTR) and non –LTR retrotransposons. Non-LTR elements have the ability to mobilise and can be further subdivided into SINE (e.g. Alu elements) and LINE (e.g. LINE1 elements). The LTR class of RTE contains endogenous retroviruses (ERVs) such as HERV-K.

TEs constitute ~45% of the human genome and can be subdivided based on their method of replication, i.e via an RNA (retrotransposable elements (RTEs)) or DNA (DNA transposons) intermediate. RTEs are further classified into long terminal repeats (LTR) or non-(LTR) elements. The later resemble integrated mRNAs and have a distinct mechanism of retrotransposition. Non-LTR RTE have also been associated with a number of diseases and can be classified as either long interspersed nuclear elements (LINEs) or short interspersed nuclear elements (SINEs)^14^. For a detailed review of the impact of RTE in the human genome and their role in disease see^15^ and an overview the classification of RE is shown in (Fig. 1).

Dysregulation of RTEs has been associated with several neurological disorders and increased expression is linked with toxicity and genomic instability^16–19^. In response to this, cells have developed various epigenetic mechanisms to ensure RTEs are tightly suppressed. However it would appear this mechanism goes awry in pathological state, with increased RTE expression being reported in conditions such as schizophrenia, Rett syndrome, Creutzfeldt-Jakob disease (CJD), ataxia telangiectasia and many cancers^20–22^. Specifically, accumulation of RTE transcripts has been described in several neurodegenerative diseases such as Alzheimer’s disease (AD) and Amyotrophic lateral sclerosis(ALS)^16–18,23^. A more relevant example of the potential toxicity of RTEs has been highlighted in a recent PD related study, which focussed on assessing the role of LINE1 (a non-LTR, LINE RTE element) in mesencephalic dopaminergic neurons. In an Engrailed-1 heterozygote model *Blaudin de The* show that LINE1 RNA upregulation correlates with increased DNA damage and cell death induced by oxidative stress. Subsequently reduction of LINE1 protects against oxidative stress *in vitro* and *in vivo*^24^.

Despite the growing body of evidence that shows that RE expression is associated with many diseases, genome-wide expression of these elements is yet to be characterized in PD. In this study, we utilized existing RNA-Seq data from the skin^12^ and blood of the same individuals and characterized RE expression in both PD patients and healthy controls. To gain novel insight into the expression of all classes of RE, unlike other methods that focus only on RTN, we utilized the well-established RepEnrich pipeline that quantifies all known RE class sequences. We report that overall REs are widely expressed in both tissues, with observed tissue-specific differences and most significantly, that two simple satellite III (repName = CATTC_n and _GAATG_n), a high-copy satellite II (repName = HSATII) and a centromeric satellite element (repName = ALR_Alpha) were differentially expressed in the blood in PD.

## Results

### Repetitive elements are widely expressed in the blood and skin

**Figure 2:**
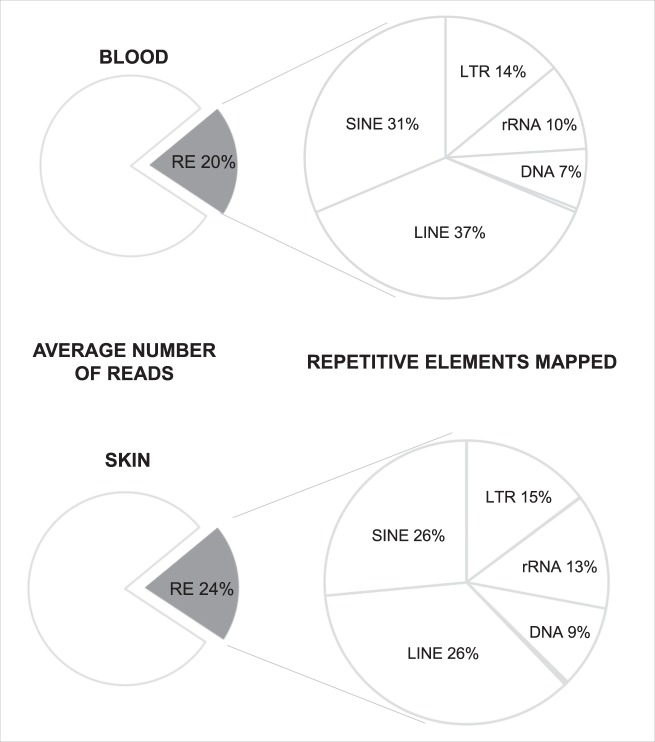
Mapped repetitive element expression in the blood and skin. Analysis of RNA-Seq data from the blood of 12 PD patients and 12 controls (with an average of 24 (blood) or 31 (skin) million reads per individual) identified 20% of reads mapping to the reference genome GRCh37/hg19, aligned to the custom built RE psuedogenome assembly used in RepEnrich. Of the reads that mapped to the RE pseudogenome assembly, in the blood on average 37.10% originated from LINE elements, 31.22% from SINE, 13.93% from LTR, 10.41% from rRNA, 6.90% from DNA and 0.44% other (satellite, snRNA, tRNA, RNA, RC, scRNA). In the skin on average 35.71% originated from LINE elements, 26.47% from SINE, 14.70% from LTR, 13.14% from rRNA, 9.41% from DNA and 0.57% other (satellite snRNA, tRNA, RNA, RC, scRNA).

Analysis of RNA-Seq data from 12 PD patients and 12 controls (with an average of 24 (blood) or 31 (skin) million reads per individual) identified that 20.3% (blood) and 23.8% (skin) of reads mapping to the reference genome GRCh37/hg19 aligned to the RE annotated in the RepeatMasker GRch37/hg19 Library (Fig. 2). No significant bias was detected in the RNA-Seq datasets by visualizing the read counts for expressed genes as principal component analysis (PCA) plots (Supplementary Figs S1–S4). REs were widely expressed, on average of the 1117 REs queried, 1086 were detected in the blood (RPKM ≥1)(97%) and 1099 in the skin. Based on RepeatMasker annotations RepEnrich output is quantified into three categories 1) expression of every RE that has a known consensus sequence in RepeatMasker, which is named as ‘subfamily’ classification (n = 1117) 2) grouping all of the REs by ‘family’ (n = 48) and 3) further sub grouping of the families by ‘class’ (n = 13). An explanation of the grouping of the RepEnrich output for expression levels is given in (Supplementary Table 1). No significant difference for relative abundance of reads originating from each of the different RE classes was observed between PD and control in the blood or in the skin (Supplementary Table 2).

Of the reads that mapped to the RE pseudogenome assembly, in the blood on average 37.10% originated from LINE elements, 31.22% from SINE, 13.93% from LTR, 10.41% from rRNA, 6.90% from DNA and 0.44% other (satellite, snRNA, tRNA, RNA, RC, scRNA). In the skin on average 35.71% originated from LINE elements, 26.47% from SINE, 14.70% from LTR, 13.14% from rRNA, 9.41% from DNA and 0.57% other (satellite, snRNA, tRNA, RNA, RC, scRNA) (Fig. 2). However when the abundance of individual members of each class were compared between the two tissues analyzed, there were significant differences in relative abundance of RE between blood and skin for the majority of the classes, highlighting previously reported tissue-specific nature of RE expression^25^.

### Satellite elements are significantly upregulated in the blood of PD patients

EdgeR analysis was applied to compare RE expression between PD patients and healthy control subjects in both the blood and skin. The edgeR-normalized pseudocounts of REs were visualized on PCA plots and no possible biases affecting the analysis were observed (Supplementary Figs S1 and S2). No significant differences in RE expression were observed comparing PD patients and healthy controls in the skin (FDR ≤ 0.01). However, upregulation of satellite REs at the class level with a log_2_FC increase of 1.93 (FDR = 7.7E-06) was identified in the blood from PD patients compared to the blood from healthy control subjects (Supplementary Table 3).

At the family level, 3 RE families were significantly differentially expressed at FDR ≤ 0.01; satellite, centr and acro (FDR = 7.88E-06, 7.88E-06, 6.39E-04 respectively), which were upregulated with a logFC increase of 2.05 for satellite, 1.84 for centr and 1.30 for acro in the blood from the PD patients (Supplementary Table 4).

**Figure 3:**
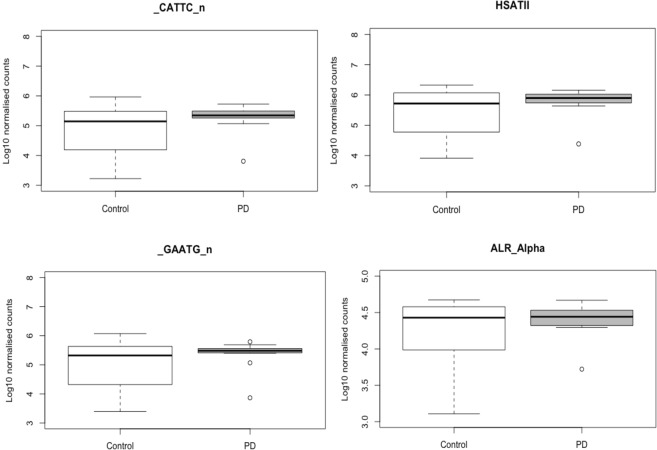
Upregulation of Satellite elements in the blood of PD patients. At the subfamily level four satellite class repetitive elements were significantly differentially expressed at FDR ≤ 0.01, two simple satellite III (repName = CATTC_n and _GAATG_n) a high-copy satellite II (repName = HSATII) and a centromeric satellite (repName = ALR_Alpha) all of which were upregulated in the blood of PD patients. Simple satellite III repeat (CATTC)n RNAs were the most significantly upregulated in the blood of PD patients (p-value = 2.27E-12) with a logFC increase of 4.40. Pericentromeric human satellite II (HSATII) repeat derived RNAs were significantly upregulated (p-value = 2.79E-12) with a logFC increase of 4.12. Simple satellite III (GAATG)n derived RNAs were upregulated in PD (p-value = 1.68E-11) with a logFC increase of 4.23. Finally, human alpha centromeric satellite (ALR_Alpha) derived RNAs were also upregulated in PD (p-value = 5.20E-08) with a 2.02 logFC increase.

At the subfamily level four specific satellite class REs were significantly differentially expressed at FDR ≤ 0.01, two simple satellite IIIs (repName = CATTC_n and _GAATG_n) a high-copy satellite II (repName = HSATII) and a centromeric satellite (repName = ALR_Alpha) all of which were upregulated in the blood of PD patients (Appendix.1. Supplementary data) (Table 1). Moreover, the expression levels of the aforementioned satellite elements displayed little inter-variability in the PD patient group when compared to healthy controls (Fig. 3).

**Table 1:** Differentially expressed repetitive elements identified in blood from PD patients.

Class	Family	RE	Description	Log FC	Log CPM	P-value	FDR
Satellite	Satellite	_CATTC_n	Simple satellite III	4.4	7.74	2.27E-12	1.56E-09
Satellite	—	HSATII	High-copy satellite II	4.12	5.69	2.79E-12	1.56E-09
Satellite	Satellite	_GAATG_n	Simple satellite III	4.23	7.32	1.68E-11	6.25E-09
Satellite	Centromeric	ALR_Alpha	171 bp satellite associate with human centromeres	2.02	8.69	5.20E-08	1.45E-05

Showing characteristics of each differentially expressed element, log FC, log CPM, p-value and FDR (≤0.01 cut off).

Simple satellite III repeat (CATTC)n RNAs were the most significantly upregulated in PD blood (p-value = 2.27E-12) with a logFC increase of 4.40. Pericentromeric human satellite II (HSATII) repeat derived RNAs were also significantly upregulated (p-value = 2.79E-12) with a logFC increase of 4.12. HSATII derived RNA should be undetectable in normal tissue and dysregulation of these elements has shown to induce genomic instability^26^. Simple satellite III (GAATG)n derived RNAs were also upregulated in the blood of PD patients compared to the blood of healthy control subjects (p-value = 1.68E-11) with a logFC increase of 4.23. Finally, human alpha centromeric satellite (ALR_Alpha) derived RNAs were also upregulated in the blood of PD patients compared to the blood of healthy control patients (p-value = 5.20E-08) with a 2.02 logFC increase (Fig. 3).

## Discussion

This study presents the first genome-wide analysis of RE expression in PD, this analysis also included a detailed characterization of the expression of REs in the blood and skin and highlights the previously reported tissue-specific nature of RE expression^25,27^. Using a stringent FDR cut-off of 0.01 we found that there was no differential expression of REs in the skin when PD patients and healthy controls were compared. However in the blood we identified that satellite elements are upregulated in PD patients and most significantly that a group of satellite elements, (repName = CATTC_n, HSATII, ALR_Alpha) which are a group of elements that have been collectively associated with genome instability^26,28^, are significantly differentially expressed.

Characterization of RNA-Seq data in the 24 subjects studied identified that REs were widely expressed and constituted 20% of all expressed transcripts in the blood and 24% of all expressed transcripts in the skin. We report no significant difference in relative abundance of global RE expression between patients versus controls for either tissue (Supplementary Table 2). Our data is in agreement with that reported by Faulkner *et al*. who performed a comprehensive RE analysis using cap analysis gene expression (CAGE) sequencing data and determined that in human tissue, on average around 20% of all CAGE tags detected were mapped to REs and overall RE expression varied significantly between tissue^25^. Our data is also supported by a recent study from our group that analyzed skin RNA-Seq data from 12 individuals with psoriasis and 12 healthy controls with the same RepEnrich pipeline and found that on average 27.5% of reads aligned to REs^27^.

In light of the recent associations between RTE dysregulation and neurodegenerative disease, more specifically the identification of LINE1 overexpression inducing death of mesencephalic dopaminergic neurons, we set out to identify if these elements were differentially expressed in the peripheral tissues of PD patients. Our analysis used a method that not only determined differential expression of the different classes of RTEs but all class of RE. Although we did not observe differentially expressed RTEs in the skin or blood of PD patients, we did identify upregulation of satellite elements in the blood of PD patients(Table 1, Fig. 3).

Overall aberrant overexpression of satellite repeats has been associated with genomic instability^29,30^. Collectively three of the upregulated satellite elements identified in this study (repName = CATTC_n and _HSATII and ALR_Alpha) have been named as a group of REs involved in genomic instability and considered to be transcriptionally silent in the genome^26^. Although the simple satellite III RE (repName = _GAATG_n) was also significantly upregulated, like many of the REs, there is a paucity of information available for the function of this RE in the literature. Interestingly, in addition to increased expression levels, a considerably small degree of variability was observed in the PD patients compared to the healthy control subjects. This could hint at a yet undetermined regulatory process that can be associated with PD pathophysiology.

Studies have shown that inducing dysregulation of genes known to silence satellite elements can promote genomic instability, which consequently can result in; growth arrest, impaired homologous recombination and spontaneous DNA breaks through this pathway. An example of this is apparent with the gene *SIRT1* (Silent information regulator-1) which is a known repressor of repetitive DNA. It has been shown in mouse ES cells that when *SIRT1* is inhibited one of the major consequences is activation of major satellite repeats and thus an increase in expressed satellite transcripts is observed^31^. This process has been associated with mitochondrial dysfunction and oxidative stress, which are common pathways that are affected during ageing and that have been implicated in PD^32^. However, it is unknown if the observed upregulation of satellite elements is a mechanistically important factor in the etiology of PD or if it is simply an indicator of pathophysiological state; thus, it will of interest in the future whether such pathways and genes are modulated in the CNS or in immune cells in PD. As shown in Fig. 3 there is a striking loss of variability in the expression of the group of satellite elements upregulated in PD, which is pattern that is not observed in other RE elements (Supplementary Fig. S5). Therefore our data indicates that overexpression of these specific satellite elements is a potential disease-specific signature in the blood for PD and highlights the need for further characterization of our model in a larger, better clinically defined, data sets to also address if there is possible association with progression of the disease.

We set out to characterise RE expression with previously published PD RNA-Seq data in response to recent studies that have associated RTE expression with neurodegenerative disease. This type of analysis has not been explored previously, mainly due to the lack of technology to do so. Using a tool that not only quantifies RTE expression but all class of REs we report that a group of satellite elements are differentially expressed and appear to have a PD specific expression signature in the blood. Although we report the first genome-wide analysis of RE expression in PD to date, our study does have a number of limitations. First, we acknowledge that the cohort size (n = 24) is particularly small. By showing that there are significant disease-specific signatures of RE expression, we hope to illustrate that RE expression should be addressed when larger PD expression data-sets are available. Another limitation is that we show through of our analysis (of the blood and skin of the same individuals) that RE expression occurs in a tissue specific manner. Thus we cannot make any presumptions that this reflects what could be happening in the brain. Although a very interesting concept, addressing RE expression in the brains of PD individuals is particularly problematic as the areas of interest will have occured alot of cell-death at time of post-mortem. Despite the literature suggesting a possible role of RTE in the brain and neurodegeneration in particular, the purpose of our present study was to look for possible differences in RE expression in readily available tissue (such as blood and skin) as a possible biomarker.

## Conclusion

In summary, the field is still far from establishing the molecular mechanisms underpinning PD and much progress is needed to develop an objective biomarker for the disease. We utilized previously existing RNA-Seq data and characterized RE expression in the blood and skin of PD patients, our rationale being, 1) recent studies have shown that disease pathology can be detected in the peripheral tissue 2) more sophisticated bioinformatic methods have provided a growing body of evidence that RTE dysregulation is associated with neurodegenerative diseases such as AD,ALS and PD^16,17,23,24^. We identified firstly, overall tissue-specific differences in RE expression as supported by the literature and secondly that a specific group of satellite elements, that have been strongly associated with epigenetic instability, displayed altered expression in the blood of patients with PD. Although we cannot comment on whether the observed upregulation of satellite elements is a mechanistically important factor in the etiology of PD or simply an indicator of a pathophysiological state, this data does support recent studies showing that PD pathogenesis can be linked with changes in the peripheral tissue^12,13^. Further characterization is required to determine the consequence of upregulation of satellite elements in PD, however our data supplies a potential novel non-invasive biomarker of the disease and its progression

## Methods

### Study participants and ethics

Blood samples were collected from 12 patients with PD (6 men and 6 women, aged 72.2 ± 9.9 years, mean ± SD), and 12 healthy control subjects (blood; 6 men and 6 women, aged 68.9 ± 6.9 years, skin). All patients fulfilled the Queen Square Brain Bank Criteria for idiopathic PD^33,34^. The mean onset age and duration of disease at sample collection were 65.5 ± 8.6 and 7.3 ± 6.3 years, respectively (Table 2). A detailed description of individuals enrolled on the skin study please refer to Planken *et al*.^12^. In brief, the median total score of the Movement Disorder Society sponsored revision of the Unified Parkinson Disease Rating Scale (MDS-UPDRS)^35^ was 65 (ranging from 22 to 159). The median disease severity assessed by the Hoehn and Yahr scale^36^ was 2.75 (ranging from 1 to 4). The median disability score assessed by the Schwab and England Activities of Daily Living Scale (SE-ADL) was 80% (ranging from 40% to 100%). None of the PD patients were current smokers and two had a history of smoking in the past.

**Table 2:** Characteristics of the PD patients: HY = Hoehn and Yahr stage; SE-ADL, Schwab and England Activities of Daily Living Scale; MMSE, Mini Mental State Examination.

Sex	Age (YR)	Disease Onset Age (YR)	Disease Duration (YR)	Disease Subtype*	HY	SE-ADL	MMSE
M	85	67	18	3	4	40	27
M	76	75	1	1	2.5	90	30
M	73	65	9	2	3	80	30
M	67	50	17	2	4	70	28
M	69	68	2	3	3	80	30
M	73	72	1	1	1	95	29
F	82	74	9	3	4	60	26
F	68	65	4	1	1.5	100	30
F	71	66	6	2	3	70	24
F	48	47	2	2	1.5	90	30
F	69	67	3	1	2.5	80	29
F	85	70	15	1	2.5	60	23

^*^1-tremor-dominant; 2-akinetic-rigid; 3-postural instability and gait disorder.

The study was approved by the Research Ethics Committee of the University of Tartu. Volunteer PD patients and healthy controls were recruited from the Department of Neurology and Neurosurgery at the University Hospital of Tartu. A signed informed consent was acquired from all subjects participating in this study.

### Library preparation

The venous blood of all study subjects was collected into Tempus Blood RNA Tubes (Thermo Fisher Scientific Inc, CA, USA). The RNA was extracted applying Tempus Spin RNA Isolation Kit (Thermo Fisher Scientific Inc, CA, USA) combined with DNase treatment (RNase-Free DNase Set, Qiagen, Hilde, Germany), according to the manufacturer’s’ protocols. The globin mRNA was removed from the extracted total RNA using GLOBINclear Kit for human (Thermo Fisher Scientific Inc, CA, USA). For the skin, one 4 mm punch-biopsy specimen was taken from non- sun-exposed skin of each subject from both study groups. All biopsy specimens were instantly frozen in liquid nitrogen and stored at −80 °C until RNA extraction. Biopsies were homogenized with Precellys24 homogenizer with the Cryolys system (Bertin Technologies). RNeasy Fibrous Tissue Mini Kit (Qiagen, Hilde, Germany) was used for total RNA extraction, according to the manufacturer’s protocol. During the purification on-column DNase I treatment was performed (Qiagen Hilde, Germany). The RNA quality was assessed using Agilent 2100 Bioanalyzer, with the RNA 6000 Nano kit (Agilent Technologies) and the quantity was evaluated with Qubit fluorometer and Qubit RNA HS Assay kit (Life Technologies). The study samples RIN ranged from 6.7–9.5 in the blood and skin samples.

### RNA sequencing

50 ng of each RNA sample was amplified with Ovation RNA-Seq System V2 Kit (NuGen Technologies Inc, CA, USA) and the output double stranded DNA was used to prepare SOLiD 5500 W System DNA fragment libraries according to manufacturer’s protocols (Thermo Fisher Scientific Inc, CA, USA). For library preparation, the barcoding adapters were used, and 12 libraries were pooled prior to sequencing. For sequencing skin samples, the SOLiD 5500 W XL with paired-end chemistry (75 bp in forward and 35 bp in reverse direction) in 6-lane mode was applied. In the case of blood samples SOLiD 5500 W XL platform with fragment sequencing chemistry (75 bp in forward directions) in 3-lane mode was used. In both cases approximately 40 million mappable reads were expected per one sample, which is enough for successful whole transcriptome expression pattern analysis.

### Read alignment and quantification

Raw color-space reads were filtered for rRNA, active tRNA, and SOLiD adaptor sequences. The remaining reads were aligned as single-end reads to the GRCh37/hg19 reference genome, while allowing multi-mapping to detect reads aligning to possible repeat sequences. To ensure secondary alignments were reported in the BAM files no mapping quality cut-off was set. LifeScope software (LifeTechnologies) with recommended settings designed for color-space read alignment and analysis was used for both mapping steps.

The number of reads aligning to known exonic gene sequences were counted and visualized by plotting the first two principal components in order to exclude the possibility of bias in the RNA-seq datasets. The base Stats package in R was used to conduct the principle component analysis (PCA)^37^. Prior to PCA, the read counts were normalized as z-scored counts per million mapped reads (CPM) values, where the standard deviation and mean were calculated separately for each gene. PCA plots are shown in (Supplementary Figs S1–S4).

To connect color-space mapping with the RepEnrich pipeline https://github.com/nskvir/RepEnrich14, the GRCh37/hg19 mapped BAM files were parsed using samtools and in-house perl scripts to separate unique and non-unique mapped reads. For uniquely mapped reads, only alignments with MAPQ ≥ 10 (in Phred scale) were retained, on average ~77% of all reads mapped to the reference genome. For multi-mapping reads, the base-space sequence was inferred from the longest alignment and these reads were converted to FASTQ format. This enabled us to convert the color-space data into suitable format for downstream analysis. RepEnrich with default parameters was applied to obtain read counts of REs RepEnrich aligns multi-mapping reads separately to a pseudogenome containing the RE loci lifted from the RepeatMasker GRch37/hg19 Library in order to more accurately infer read counts which reflect the abundance of expressed RE.

### Analysis of differentially expressed repetitive elements

**Figure 4:**
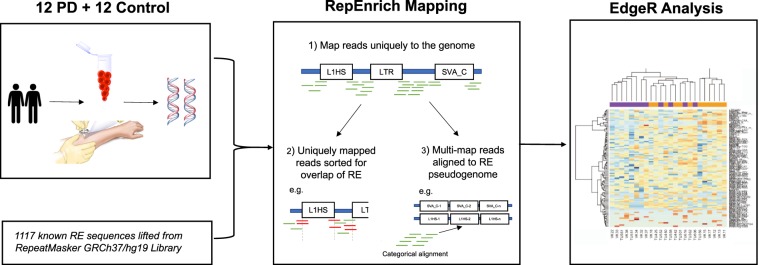
RepEnrich Workflow. RNA-Sequencing data was obtained from blood and skin of 12 PD patients and 12 healthy controls. A RE pseudogenome assembly was constructed by concatenating the genomic sequence for the 1117 RE elements from the ReRCh37/hg19 Library. Reads were mapped using the RepEnrich pipeline and differential RE expression was identified following EdgeR analysis.

The R package edgeR (Robinson *et al*. 2010) was used to identify differentially expressed REs at sub-family, family and class level between the case and control in the blood and skin. The EdgeR package uses a negative binomial model to infer the significance of differential read counts.The RE overall library sizes were used for normalization of read counts for each sample. Prior to differential expression testing, the edgeR-normalized RE pseudocounts were visualized by plotting the first two principal components in order to check for potential bias in the RE counts data. The generalized linear model approach of EdgeR was then applied to compare PD to control. The workflow used for the RepEnrich analysis is outlined in (Fig. 4).

### Ethics approval and consent to participate

The study was conducted in accordance with the Declaration of Helsinki and approval was granted by the Tartu University Ethics Committee. Informed consent was obtained from all patients and controls.

## Supplementary information

Supplementary Material

Supplementary data

## Data Availability

The data supporting the conclusions of this article and raw counts from the RepEnrich output are included within this article and its supplementary information.
