# Supplementary material for: Analysis of repetitive element expression in the blood and skin of patients with Parkinson’s disease identifies differential expression of satellite elements
Source: Sci Rep. 2019 Mar 13;9:4369. doi: 10.1038/s41598-019-40869-z (PMC6416352; doi:10.1038/s41598-019-40869-z)
Supplement: Supplementary file 1 — Supplementary Material [file 41598_2019_40869_MOESM1_ESM.docx]

**Analysis of repetitive element expression in the blood and skin of patients with Parkinson’s disease identifies differential expression of satellite elements**

Kimberley J. Billingsley^1*^, Freddy Lättekivi^2*^, Anu Planken^3^, Ene Reimann^2^, Lille Kurvits^4^, Liis Kadastik-Eerme^3,^Kristjan M. Kasterpalu^5^, Vivien J. Bubb^1^, John P. Quinn^1^, Sulev Kõks^6$^, Pille Taba^3^

**Supplementary Materials:**

**Table S1:** Explanation of RepEnrich output for expression levels. Based on RepeatMasker data the output is quantified in three catergories 1) expression of every RE that has a known consensus sequence in RepeatMasker (subfamily) (n= 1117) 2) grouping all of the RE by family (n=48) and 3) grouping the families further into classes (n= 13).

| **RE Subdfamily(n=1117)** | **RE family (n=48)** | **RE class (n=13)** |
| --- | --- | --- |
| Every known RE consensus sequence in RepeatMasker | satellite,centr,acro,telo | Satellite |
|  | RNA | RNA |
|  | Helitron | RC |
|  | scRNA | scRNA |
|  | rRNA | rRNA |
|  | tRNA | tRNA |
|  | srpRNA | srpRNA |
|  | ERVL,ERVL-MaLR,ERV1,Gypsy,LTR,ERVK,ERV1?,Deu,Gypsy?,ERVL? | LTR |
|  | Other | Other |
|  | Dong-R4,L1,CR1,RTE-BovB,L2,L1?,RTE-X | LINE |
|  | Alu,MIR | SINE |
|  | TcMar-Mariner,TcMar?,hAT-Tip100,DNA,hAT-Charlie,hAT-Tip100?,hAT-Blackjack,PiggyBac?,hAT?,TcMar-Tc2,TcMar-Pogo,TcMar,PiggyBac,TcMar-Tigger,hAT,Merlin,MULE-MuDR | DNA |
|  | snRNA | snRNA |

**Table S2**. Relative abundance (%) of repetitive element classes expressed in the blood and skin of PD patients and healthy controls. Showing average per study group, p-value for two- sided t-test comparison and overall average per tissue. * indicates significant difference in relative abundance of each repetitive element class when skin and blood we compared in PD and control combined (p ≤ 0.01).

|  | **BLOOD** | | | | **SKIN** | | | |
| --- | --- | --- | --- | --- | --- | --- | --- | --- |
| RE class | % OF RE: CONTROL | % OF RE: PD | P-VALUE  (T-TEST) | AVERAGE | % OF RE: CONTROL | % OF RE: PD | P-VALUE  (T-TEST) | AVERAGE |
| *srpRNA | 0.02 | 0.03 | 0.31 | **0.03** | 0.04 | 0.04 | 0.11 | **0.04** |
| *LTR | 13.81 | 14.06 | 0.38 | **13.93** | 14.75 | 14.65 | 0.67 | **14.70** |
| SATELLITE | 0.15 | 0.07 | 0.15 | **0.11** | 0.10 | 0.08 | 0.26 | **0.09** |
| *rRNA | 10.96 | 9.87 | 0.48 | **10.41** | 13.16 | 13.11 | 0.96 | **13.14** |
| *DNA | 6.86 | 6.94 | 0.48 | **6.90** | 9.49 | 9.33 | 0.10 | **9.41** |
| *snRNA | 0.04 | 0.04 | 0.56 | **0.04** | 0.08 | 0.07 | 0.01 | **0.07** |
| tRNA | 0.06 | 0.05 | 0.29 | **0.05** | 0.06 | 0.07 | 0.18 | **0.07** |
| *RNA | 0.06 | 0.03 | 0.07 | **0.04** | 0.03 | 0.05 | 0.03 | **0.04** |
| OTHER | 0.10 | 0.10 | 0.92 | **0.10** | 0.22 | 0.21 | 0.39 | **0.22** |
| *RC | 0.04 | 0.04 | 0.42 | **0.04** | 0.03 | 0.03 | 0.30 | **0.03** |
| LINE | 36.79 | 37.42 | 0.47 | **37.10** | 35.41 | 36.01 | 0.42 | **35.71** |
| *SINE | 31.12 | 31.32 | 0.72 | **31.22** | 26.60 | 26.34 | 0.58 | **26.47** |
| *scRNA | 0.04 | 0.04 | 0.63 | **0.04** | 0.02 | 0.02 | 0.05 | **0.02** |

**Table S3:** Analysis of RE in the blood class-level pairwise comparison with EdgeR.

| **RE Class** | **logFC** | **logCPM** | **p-value** | **FDR** |
| --- | --- | --- | --- | --- |
| Satellite | 1.93 | 10.10 | 5.44E-07 | 7.07E-06 |
| RNA | 0.50 | 7.94 | 0.04 | 0.26 |
| RC | -0.13 | 8.69 | 0.07 | 0.30 |
| scRNA | -0.15 | 8.69 | 0.09 | 0.30 |
| rRNA | 0.19 | 16.68 | 0.36 | 0.74 |
| tRNA | 0.21 | 9.10 | 0.37 | 0.74 |
| srpRNA | 0.13 | 8.03 | 0.40 | 0.74 |
| LTR | -0.04 | 17.09 | 0.59 | 0.88 |
| Other | 0.10 | 9.92 | 0.61 | 0.88 |
| LINE | -0.01 | 18.50 | 0.69 | 0.89 |
| SINE | 0.00 | 18.25 | 0.84 | 0.97 |
| DNA | 0.01 | 16.07 | 0.97 | 0.97 |
| snRNA | 0.00 | 8.76 | 0.97 | 0.97 |

**Table S4**: Analysis of RE in the blood family-level pairwise comparison with EdgeR.

| **RE Family** | **logFC** | **logCPM** | **PValue** | **FDR** |
| --- | --- | --- | --- | --- |
| Satellite | 2.05 | 9.32 | 2.88E-07 | 7.88E-06 |
| centr | 1.84 | 8.81 | 3.28E-07 | 7.88E-06 |
| acro | 1.28 | 2.60 | 3.9958E-05 | 6.39E-04 |
| RNA | 0.48 | 7.93 | 0.02 | 0.28 |
| telo | 0.20 | 3.56 | 0.06 | 0.62 |
| TcMar-Mariner | -0.07 | 10.90 | 0.12 | 0.79 |
| ERVL | -0.10 | 14.95 | 0.15 | 0.79 |
| scRNA | -0.19 | 8.71 | 0.18 | 0.79 |
| TcMar? | -0.14 | 6.81 | 0.19 | 0.79 |
| hAT-Tip100 | -0.07 | 12.28 | 0.19 | 0.79 |
| ERVL-MaLR | -0.06 | 15.54 | 0.20 | 0.79 |
| DNA | 0.11 | 6.49 | 0.20 | 0.79 |
| Helitron | -0.17 | 8.70 | 0.22 | 0.80 |
| Dong-R4 | -0.11 | 6.24 | 0.25 | 0.83 |
| ERV1 | -0.08 | 15.74 | 0.26 | 0.83 |
| srpRNA | 0.12 | 8.02 | 0.33 | 0.93 |
| Gypsy | -0.08 | 10.24 | 0.34 | 0.93 |
| rRNA | 0.17 | 16.67 | 0.35 | 0.93 |
| ERVL? | -0.10 | 9.08 | 0.38 | 0.93 |
| tRNA | 0.13 | 9.89 | 0.46 | 0.93 |
| hAT-Charlie | -0.03 | 15.18 | 0.47 | 0.93 |
| L1 | -0.05 | 18.18 | 0.48 | 0.93 |
| hAT-Tip100? | 0.06 | 6.73 | 0.52 | 0.93 |
| hAT-Blackjack | 0.06 | 10.49 | 0.53 | 0.93 |
| LTR | -0.05 | 8.56 | 0.57 | 0.93 |
| Alu | -0.04 | 18.00 | 0.59 | 0.93 |
| CR1 | 0.02 | 12.82 | 0.60 | 0.93 |
| MIR | 0.03 | 15.61 | 0.60 | 0.93 |
| ERVK | 0.03 | 11.65 | 0.61 | 0.93 |
| Other | 0.06 | 9.91 | 0.61 | 0.93 |
| PiggyBac? | 0.06 | 3.59 | 0.65 | 0.93 |
| hAT? | 0.04 | 4.91 | 0.65 | 0.93 |
| TcMar-Tc2 | 0.03 | 9.86 | 0.74 | 0.93 |
| TcMar-Pogo | 0.15 | -0.46 | 0.75 | 0.93 |
| TcMar | 0.04 | 4.48 | 0.75 | 0.93 |
| PiggyBac | -0.03 | 8.31 | 0.75 | 0.93 |
| RTE-BovB | 0.03 | 5.36 | 0.77 | 0.93 |
| TcMar-Tigger | -0.01 | 14.36 | 0.78 | 0.93 |
| L2 | -0.01 | 15.99 | 0.78 | 0.93 |
| snRNA | -0.03 | 8.76 | 0.78 | 0.93 |
| L1? | -0.06 | 1.98 | 0.79 | 0.93 |
| ERV1? | 0.02 | 6.48 | 0.86 | 0.95 |
| Deu | 0.02 | 8.23 | 0.87 | 0.95 |
| Gypsy? | 0.02 | 8.74 | 0.87 | 0.95 |
| hAT | 0.01 | 8.84 | 0.91 | 0.95 |
| Merlin | -0.01 | 3.17 | 0.91 | 0.95 |
| MULE-MuDR | 0.01 | 8.42 | 0.95 | 0.96 |
| RTE-X | 0.00 | 10.64 | 0.96 | 0.96 |

**Supplementary Figures**:

**
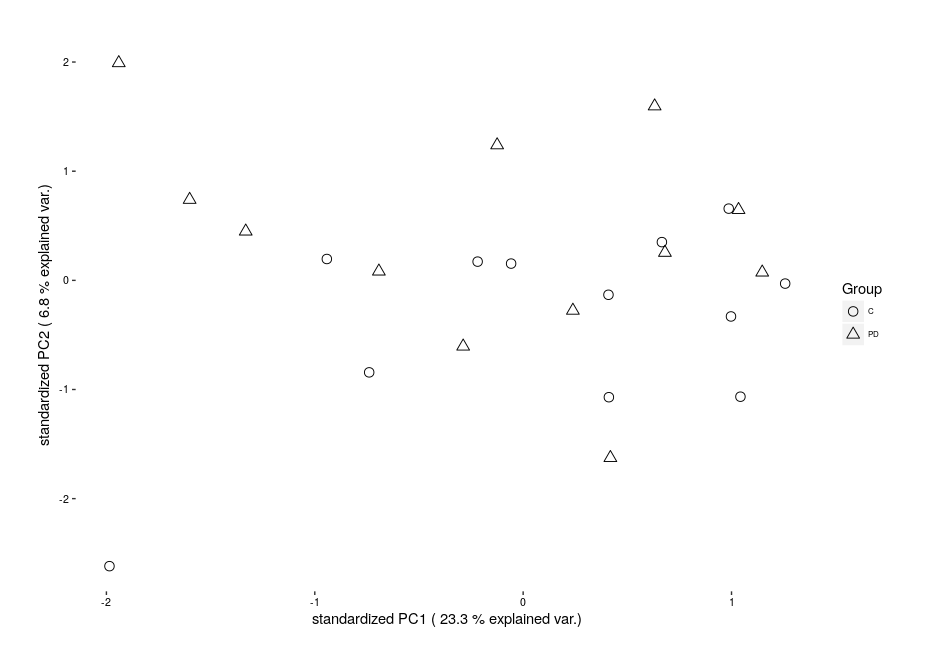
**

**Figure S1:** Principle component analysis (PCA) plot of first two principle components calculated from the read counts data of known exonic gene sequences in the hg19 reference genome in the blood dataset. Read counts were normalized as z-scored counts per million mapped reads (CPM) values, where the standard deviation and mean were calculated separately for each gene. Samples belonging to Parkinson’s disease (PD) and healthy control (C) are displayed as different symbols.


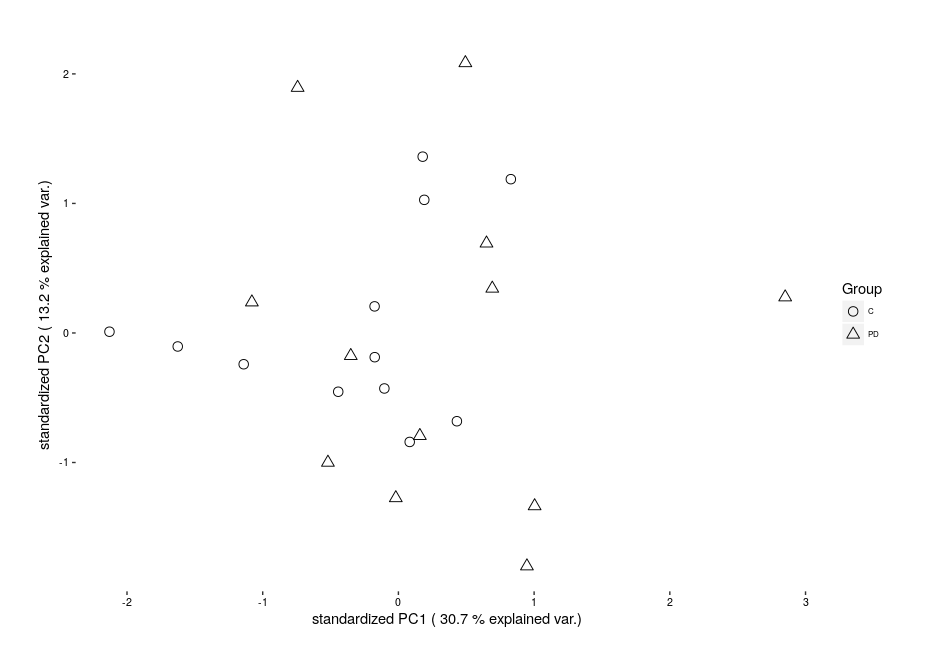


**Figure S2:** Principle component analysis (PCA) plot of first two principle components calculated based on the pseudocounts of repetitive elements in the hg19 reference genome in the blood dataset. Raw read counts were transformed into pesudocounts using edgeR normalization factors. For PCA analysis, pseudocounts were further normalized by converting them into z-scored counts per million mapped reads (CPM) values, where the standard deviation and mean were calculated separately for each gene. Samples belonging to Parkinnson’s disease (PD) and healthy control (C) are displayed as different symbols.


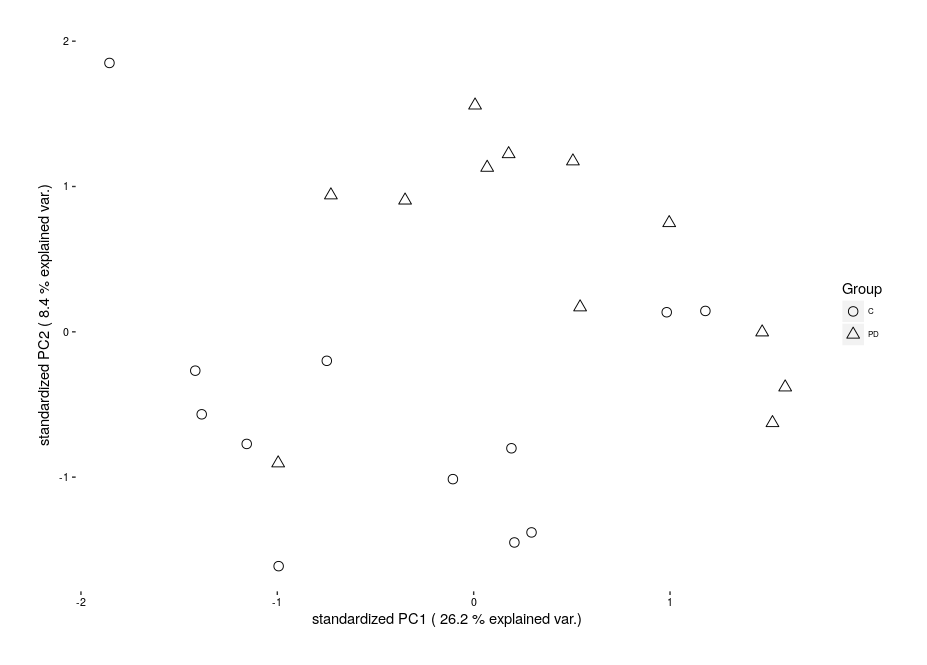


**Figure S3:** Principle component analysis (PCA) plot of first two principle components calculated from the read counts data of known exonic gene sequences in the hg19 reference genome in the skin dataset. Read counts were normalized as z-scored counts per million mapped reads (CPM) values, where the standard deviation and mean were calculated separately for each gene. Samples belonging to Parkinnson’s disease (PD) and healthy control (C) are displayed as different symbols.


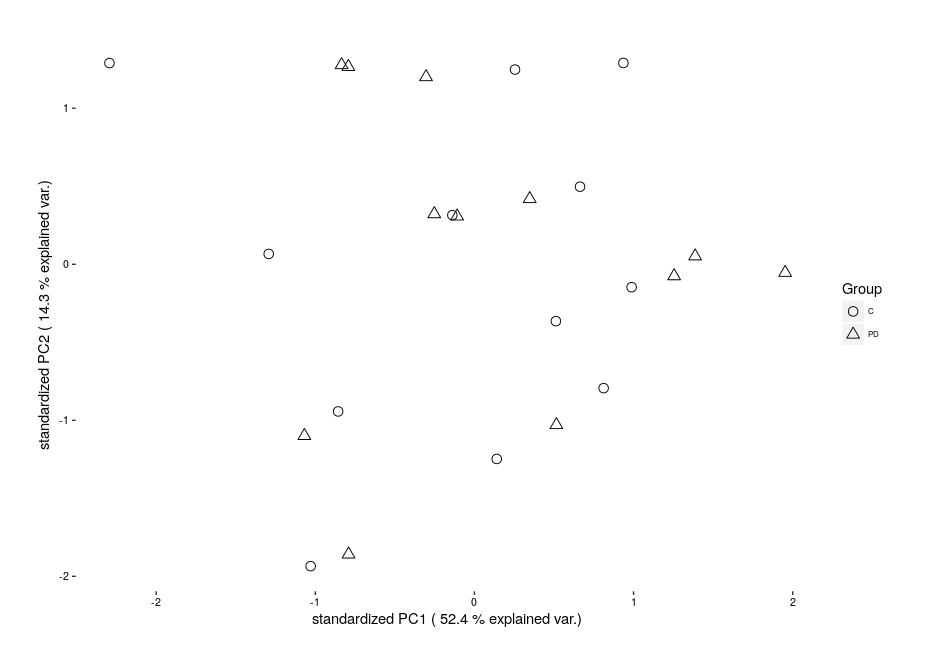


**Figure S4:** Principle component analysis (PCA) plot of first two principle components calculated based on the pseudocounts of repetitive elements in the hg19 reference genome in the skin dataset. Raw read counts were transformed into pesudocounts using edgeR normalization factors. For PCA analysis, pseudocounts were further normalized by converting them into z-scored counts per million mapped reads (CPM) values, where the standard deviation and mean were calculated separately for each gene. Samples belonging to Parkinson’s disease (PD) and healthy control (C) are displayed as different symbols.

**Figure S5:** Following the lack of observed variability in the upregulated satellite elements in PD individuals, four non-significantly expressed , randomly selected REs were plotted. This included 3 other non-significant REs (Repname =L1HS, SVA_F, MER11B) and another satellite elements (Repname = HSAT5). We did not observed this lack of variability in PD individuals in the non-significant individuals, further strengthening the point that this is a disease-specific signature only observed with this particular set of upregulated elements.
